# Improved Child Feces Management Mediates Reductions in Childhood Diarrhea from an On-Site Sanitation Intervention: Causal Mediation Analysis of a Cluster-Randomized Trial in Rural Bangladesh

**DOI:** 10.1007/s44197-024-00210-y

**Published:** 2024-03-20

**Authors:** Jesse D. Contreras, Mahfuza Islam, Andrew Mertens, Amy J. Pickering, Benjamin F. Arnold, Jade Benjamin-Chung, Alan E. Hubbard, Mahbubur Rahman, Leanne Unicomb, Stephen P. Luby, John M. Colford, Ayse Ercumen

**Affiliations:** 1 Department of Epidemiology, School of Public Health, University of Michigan, Ann Arbor, MI 48103 USA; 2 Department of Environmental Health Sciences, School of Public Health, University of California, Berkeley, Berkeley, CA 94720 USA; 3 Division of Epidemiology and Biostatistics, School of Public Health, University of California, Berkeley, Berkeley, CA 94720 USA; 4 Department of Civil and Environmental Engineering, University of California, Berkeley, Berkeley, CA 94720 USA; 5 Chan Zuckerberg Biohub, San Francisco, CA 94158 USA; 6 Francis I. Proctor Foundation, University of California, San Francisco, San Francisco, CA 94158 USA; 7 Department of Epidemiology and Population Health, Stanford University, Palo Alto, CA 94304 USA; 8 Environmental Health and WASH, Health Systems and Population Studies Division, International Centre for Diarrhoeal Disease Research Bangladesh (icddr,b), Dhaka, 1212 Bangladesh; 9 Woods Institute for the Environment, Stanford University, Stanford, CA 94305 USA; 10 Department of Forestry and Environmental Resources, North Carolina State University, Raleigh, NC 27695 USA; 11 Jordan Hall Addition 2225, Raleigh, NC 27606 USA

**Keywords:** Sanitation, Latrine, Child feces, Intervention, Diarrheal disease, Mediation

## Abstract

**Background:**

The WASH benefits Bangladesh trial multi-component sanitation intervention reduced diarrheal disease among children < 5 years. Intervention components included latrine upgrades, child feces management tools, and behavioral promotion. It remains unclear which components most impacted diarrhea.

**Methods:**

We conducted mediation analysis within a subset of households (n = 720) from the sanitation and control arms. Potential mediators were categorized into indicators of latrine quality, latrine use practices, and feces management practices. We estimated average causal mediation effects (ACME) as prevalence differences (PD), defined as the intervention’s effect on diarrhea through its effect on the mediator.

**Results:**

The intervention improved all indicators compared to controls. We found significant mediation through multiple latrine use and feces management practice indicators. The strongest mediators during monsoon seasons were reduced open defecation among children aged < 3 and 3–8 years, and increased disposal of child feces into latrines. The strongest mediators during dry seasons were access to a flush/pour-flush latrine, reduced open defecation among children aged 3–8 years, and increased disposal of child feces into latrines. Individual mediation effects were small (PD = 0.5–2 percentage points) compared to the overall intervention effect but collectively describe significant mediation pathways.

**Discussion:**

The effect of the WASH Benefits Bangladesh sanitation intervention on diarrheal disease was mediated through improved child feces management and reduced child open defecation. Although the intervention significantly improved latrine quality, relatively high latrine quality at baseline may have limited benefits from additional improvements. Targeting safe child feces management may increase the health benefits of rural sanitation interventions.

**Supplementary Information:**

The online version contains supplementary material available at 10.1007/s44197-024-00210-y.

## Introduction

Access to safely managed sanitation is believed to be crucial for reducing enteric pathogen transmission and diarrheal disease among children. However, only three out of nine controlled, latrine-based interventions conducted to date have achieved reductions in diarrheal disease among children [1, 2]. Of those three, one study attributed its effect, at least in part, to uncontrolled confounding between urban and rural communities [3] and one found that reductions in diarrhea were not sustained beyond 3 months after intervention implementation [2]. The WASH Benefits Bangladesh trial appears unique as a latrine-based sanitation intervention that has rigorously measured a sustained reduction in diarrheal disease among children [4, 5]. The intervention reduced the prevalence of caregiver-reported diarrheal disease among children under five for at least 3.5 years after implementation [5] and also reduced the prevalence of infection with *Giardia* [6] and *Trichuris trichiura* [7] in children.

Multiple factors could potentially explain the WASH Benefits Bangladesh intervention’s unique effects on child health. The sanitation intervention had multiple components, including cost-free provision of improved latrines (or upgrades to pre-existing latrines) and tools for child feces management (potties and scoops), as well as frequent in-home visits for behavioral promotion [4]. With cost-free provision and ongoing promotion, WASH Benefits measured the efficacy of sanitation hardware under ideal circumstances in which cost was not a barrier to access and a sustainable local sanitation system was not required. This design differs from commonly employed interventions designed to increase demand for sanitation without provision of facilities, such as community-led total sanitation (CLTS) interventions, which have not resulted in sustained diarrheal reductions in controlled trials [1, 2]. The WASH Benefits intervention also included education and promotion for safe management of child and animal feces, along with direct provision of necessary tools for feces management. Although child feces management has been studied extensively in recent years [8], it is rarely targeted within sanitation interventions and there is little information on the independent health effects of safe child feces management [9]. Thus, the WASH Benefits trial had several distinct features compared to historical sanitation interventions, and its design may have contributed to its unique effects on diarrhea. However, WASH Benefits was also a multi-site trial conducted in Bangladesh and Kenya with mixed results across sites [10]. Despite employing the same study design and intervention in both sites, with some tailoring to the local contexts, reductions in diarrhea were found in Bangladesh alone and not in Kenya [11]. These mixed results suggest additional context-specific mechanisms responsible for the observed effects, such as more prevalent sanitation norms at baseline or higher uptake of intervention components in Bangladesh [4, 11].

Understanding the WASH Benefits Bangladesh intervention’s unique effects on diarrheal disease can provide key information for the development of effective interventions targeted to specific populations and their existing sanitation contexts. In this analysis, we aimed to identify the specific causal pathways through which this multi-component intervention impacted diarrheal disease using causal mediation analysis [12]. The analysis was carried out within a longitudinal substudy in the sanitation and control arms of the WASH Benefits Bangladesh trial conducted between 1 and 3.5 years after intervention implementation. Specifically, we estimated mediated effects between intervention allocation and diarrheal disease through indicators of latrine quality, latrine use practices, and feces management practices.

## Materials and Methods

### Study Design

#### WASH Benefits Bangladesh

WASH Benefits Bangladesh was a cluster-randomized controlled trial of water, sanitation, hygiene, and nutrition interventions (NCT01590095). The trial targeted households with a pregnant woman in their first or second trimester. Households were generally part of multifamily compounds that share a courtyard. The trial primarily targeted the household with the enrolled pregnant woman (the index household) but included the entire compound. Participating compounds were grouped into clusters of 6–8 spatially adjacent compounds, with at least 15 min walking distance between clusters to reduce spillover effects. Eight adjacent clusters formed a study block, and each cluster within a block was randomly assigned to one of six intervention arms (water; sanitation; handwashing; water, sanitation, and handwashing (WSH); nutrition; or WSH and nutrition) or a double-sized control arm. Thus, clusters were geographically matched within blocks.

The sanitation intervention comprised double-pit pour flush latrines, a sani-scoop for the removal of child and animal feces, a children’s potty, and in-personal behavioral promotion on product use and maintenance. The intervention was tailored to local preferences through pre-trial piloting [13]. Any latrines in the compound that did not have a slab or functional water seal or that failed to prevent surface runoff of feces were upgraded. A new latrine was provided to each household in the compound that did not have their own latrine, which was true for 79% of index households. A sani-scoop was provided to all households in the compound and potties were provided to all households with children < 3 years. Interventions consisted of visible components and could not be masked to participants or field staff. Behavior promotion primarily targeted the index household, but all households in the compound were invited to participate. Promoters did not visit compounds in the control arm.

#### Substudy Design

We conducted a longitudinal substudy within the WASH Benefits Bangladesh parent trial. We randomly sampled half of the participating households from the sanitation cluster and from one of two control clusters within each study block, maintaining geographic matching. We visited households eight times, approximately 4 months apart, between 1 and 3.5 years after the intervention was initiated. During each visit, field staff administered a structured survey with compound members, including child health symptoms reported by the primary caregiver of each child < 5 years in the compound, sanitation and defecation behaviors for members of the index household, and how child feces were handled for the index child (children in-utero at enrollment). Field staff also observed each latrine and sanitary conditions in the compound at each visit.

### Ethics

Written informed consent was collected from the primary caregiver of enrolled children in the local language (Bengali). Human subjects committees at the International Centre for Diarrhoeal Disease Research, Bangladesh (icddr,b) (PR-11063), University of California, Berkeley (2011-09-3652), and Stanford University (25863) approved the study protocol.

### Data Analysis

#### Outcome Variable

We followed a pre-registered data analysis plan (available at: https://osf.io/9eaku/) to assess mediating factors between the sanitation intervention and child diarrhea. The primary outcome was the prevalence of caregiver-reported diarrheal disease in the past 7 days among children < 5 years living in index households. We defined diarrhea as at least three loose stools within 24 h or at least one stool with blood, consistent with the definition used in the parent trial [4]. In a previous analysis of effect modification, we found that intervention effects on diarrheal disease were exclusive to children living in the index household that was primarily targeted by the intervention; children in index households had 26% lower diarrhea prevalence in the sanitation arm compared to controls but there was no effect among children living in other households within the compound [5]. Therefore, we restricted the mediation analysis to children living in index households.

#### Potential Mediators

Potential mediators were variables hypothesized as causally affected by the intervention that in turn impacted the prevalence of diarrheal disease. We considered potential mediators within three categories: (i) latrine quality indicators, (ii) latrine use practices, and (iii) feces management practices, with multiple individual variables within each category (Table 1). Latrine quality indicators were mostly observed directly by study staff, while latrine use and feces management indicators were typically reported by participants. While we expect myriad upstream variables to affect latrine use and feces management behaviors, such as cultural norms and psychosocial factors (e.g., self-efficacy) [14, 15], this study was not designed to investigate mediation by such upstream factors. Instead, we focused on end-point metrics of latrine quality, latrine use, and feces management to illuminate which specific intervention components drove the intervention effects on child diarrhea. We collected repeated measurements of potential mediators over eight survey rounds, except for the age of the latrine primarily used by the index households, which was recorded during the first two rounds only.

**Table 1:** Effect of the intervention on potential mediators by season (mediation analysis step one)

Mediator (modeled as outcome variable)	Monsoon season			Dry season		
	Sanitation arm n (%) or median (range)	Control arm n (%) or median (range)	Effect estimate^a^ sanitation versus control arm (95% CI)	Sanitation arm n (%) or median (range)	Control arm n (%) or median (range)	Effect estimate^a^ sanitation versus control arm (95% CI)
*Latrine quality indicators*						
Primary latrine used by index household is hygienic	1893 (97.1%)	1406 (76.1%)	1.28 (1.24, 1.31)	1301 (98.5%)	994 (77.2%)	1.28 (1.24, 1.32)
Primary latrine is flush or pour-flush	1937 (99.3%)	1241 (67.4%)	1.48 (1.43, 1.53)	1310 (99.2%)	823 (64.2%)	1.56 (1.49, 1.62)
Primary latrine has functional water seal	1867 (96.3%)	712 (56.2%)	1.72 (1.63, 1.81)	1275 (97.3%)	544 (59.4%)	1.65 (1.56, 1.75)
Primary latrine has slab	1947 (99.8%)	1782 (96.4%)	1.04 (1.03, 1.05)	1321 (100%)	1236 (96%)	1.04 (1.03, 1.05)
Primary latrine has improved floor materials	1947 (99.8%)	1782 (96.5%)	1.04 (1.03, 1.05)	1321 (100%)	1238 (96.2%)	1.04 (1.03, 1.05)
Age of primary latrine, 1 year increase (recorded in rounds 1–2 only)	1 (0, 27)	4 (0, 24)	0.45 (0.38, 0.54)	2 (0, 30)	4 (0, 40)	0.45 (0.39, 0.51)
*Latrine use practices*						
Primary latrine is shared with other households	355 (18.2%)	924 (50%)	0.36 (0.32, 0.40)	225 (17%)	674 (52.4%)	0.32 (0.28, 0.37)
Number of households primary latrine shared with	0 (0, 5)	0.5 (0, 8)	0.31 (0.27, 0.34)	0 (0, 5)	2 (0, 7)	0.28 (0.24, 0.32)
Number of people who use primary latrine	5 (0, 20)	6 (0, 57)	0.74 (0.72, 0.76)	5 (1, 18)	6 (0, 23)	0.71 (0.68, 0.73)
Visible feces in pit of primary latrine	751 (38.8%)	767 (60.3%)	0.62 (0.58, 0.66)	486 (37.1%)	507 (55.3%)	0.66 (0.60, 0.72)
Primary latrine appears to be used	1943 (99.6%)	1831 (99.1%)	1.01 (1.00, 1.01)	1311 (99.2%)	1281 (99.5%)	1.00 (0.99, 1.00)
Feces on floor in primary latrine	32 (1.6%)	159 (8.6%)	0.19 (0.13, 0.28)	36 (2.7%)	174 (13.5%)	0.20 (0.14, 0.28)
Time since primary latrine last cleaned, days	2 (0, 90)	6 (0, 180)	0.34 (0.31, 0.38)	2 (0, 45)	6 (0, 90)	0.33 (0.30, 0.37)
Men in household always/usually use latrine for defecation	1850 (97.7%)	1636 (93.2%)	1.05 (1.03, 1.06)	1260 (98.4%)	1129 (93.8%)	1.05 (1.03, 1.07)
Women in household always/usually use latrine for defecation	1933 (99.1%)	1823 (98.6%)	1.00 (1.00, 1.01)	1309 (99.2%)	1283 (99.7%)	0.99 (0.99, 1.00)
Children 8–15 in household always/usually use latrine for defecation	954 (96%)	824 (94.6%)	1.02 (1.00, 1.04)	619 (97.3%)	553 (94.7%)	1.03 (1.01, 1.05)
Children 3–8 in household always/usually use latrine for defecation	973 (77.8%)	674 (58.1%)	1.34 (1.27, 1.42)	610 (77.5%)	445 (57.1%)	1.36 (1.27, 1.47)
Children under 3 in household always/usually use latrine for defecation	100 (6.1%)	81 (5.2%)	1.18 (0.89, 1.58)	92 (8%)	72 (6.3%)	1.32 (0.98, 1.77)
Human feces observed in courtyard	15 (0.8%)	21 (1.1%)	0.80 (0.41, 1.55)	15 (1.1%)	25 (1.9%)	0.65 (0.36, 1.17)
Men in household ever practice open defecation	26 (1.4%)	182 (10%)	0.14 (0.09, 0.21)	14 (1.1%)	129 (10.5%)	0.11 (0.06, 0.20)
Women in household ever practice open defecation	1 (0.1%)	85 (4.4%)	0.01 (0.00, 0.08)	3 (0.2%)	40 (3%)	0.10 (0.02, 0.42)
Children 8–15 in household ever practice open defecation	7 (0.7%)	85 (9.8%)	0.07 (0.03, 0.15)	3 (0.5%)	59 (10.4%)	0.06 (0.02, 0.18)
Children 3–8 in household ever practice open defecation	307 (24.7%)	659 (55.1%)	0.45 (0.40, 0.50)	186 (23.8%)	397 (50.4%)	0.48 (0.42, 0.56)
Children under 3 in household ever practice open defecation	1316 (79.9%)	1529 (93.3%)	0.86 (0.83, 0.88)	887 (75.9%)	1066 (90.9%)	0.83 (0.80, 0.86)
*Feces management practices*						
Primary latrine last emptied by a professional	50 (65.8%)	272 (68.2%)	0.94 (0.79, 1.13)	21 (65.6%)	141 (61%)	1.11 (0.85, 1.46)
New latrine built within compound since previous visit	52 (3.4%)	107 (7.2%)	0.48 (0.34, 0.66)	101 (7.8%)	107 (8.3%)	0.96 (0.74, 1.25)
What was done with index child’s most recent feces			9.98 (8.61, 11.59)^b^			10.34 (8.66, 12.40)^b^
Left there in courtyard	43 (2.2%)	128 (6.7%)		49 (3.7%)	103 (7.8%)	
Thrown into environment or garbage	423 (21.7%)	1327 (69.4%)		267 (20.1%)	920 (70%)	
Used or put into latrine	1483 (76.1%)	457 (23.9%)		1010 (76.2%)	291 (22.1%)	
How was index child’s feces handled						
Used hands, cloth, paper, leaves, straw	173 (11.1%)	728 (47.1%)	Ref.	112 (10.6%)	449 (41.7%)	Ref.
Used potty, sani-scoop, or other instrument	1382 (88.9%)	819 (52.9%)	1.68 (1.60, 1.76)	941 (89.4%)	627 (58.3%)	1.54 (1.46, 1.63)
Owns child potty	1904 (97.3%)	533 (27.8%)	3.52 (3.28, 3.79)	1312 (98.8%)	410 (31.2%)	3.24 (2.99, 3.51)
Potty use by index child in past week			37.46 (31.10, 45.31)^b^			31.94 (25.72, 39.87)^b^
Don’t own or never use	77 (5.2%)	1412 (83.6%)		49 (4.7%)	929 (80.6%)	
Used to use potty, but no longer	7 (0.5%)	12 (0.7%)		8 (0.8%)	8 (0.7%)	
Less than half the time	232 (15.5%)	45 (2.7%)		197 (18.9%)	55 (4.8%)	
More than half the time	817 (54.8%)	134 (7.9%)		537 (51.6%)	89 (7.7%)	
Every time	359 (24.1%)	87 (5.1%)		249 (23.9%)	72 (6.2%)	
Owns sani-scoop	1947 (99.5%)	1689 (88.2%)	1.13 (1.11, 1.15)	1323 (99.6%)	1163 (88.4%)	1.12 (1.10, 1.15)
Sani-scoop or other tool used to pick up child feces	1070 (54.7%)	1370 (71.5%)	0.76 (0.72, 0.80)	687 (51.7%)	951 (72.3%)	0.69 (0.65, 0.74)
Sani-scoop or other tool used to pick up animal feces	1808 (92.4%)	1414 (73.8%)	1.25 (1.22, 1.29)	1238 (93.2%)	962 (73.2%)	1.27 (1.23, 1.32)
Number of piles of poultry feces in courtyard			1.08 (0.96, 1.21)^b^			0.99 (0.86, 1.14)^b^
0	197 (10.1%)	231 (12.1%)		87 (6.6%)	91 (6.9%)	
1–3	627 (32.1%)	582 (30.4%)		337 (25.4%)	307 (23.3%)	
4–10	692 (35.4%)	712 (37.2%)		471 (35.5%)	504 (38.3%)	
> 10	440 (22.5%)	391 (20.4%)		433 (32.6%)	413 (31.4%)	
Number of piles of cattle feces in courtyard			0.81 (0.70, 0.93)^b^			0.86 (0.72, 1.02)^b^
0	1463 (74.8%)	1358 (70.9%)		987 (74.3%)	942 (71.6%)	
1–2	201 (10.3%)	204 (10.6%)		137 (10.3%)	129 (9.8%)	
3–10	223 (11.4%)	261 (13.6%)		135 (10.2%)	180 (13.7%)	
> 10	69 (3.5%)	93 (4.9%)		69 (5.2%)	64 (4.9%)	
Number of piles of goat/sheep feces in courtyard			0.88 (0.76, 1.03)^b^			0.86 (0.70, 1.05)^b^
0	1531 (78.3%)	1466 (76.5%)		1115 (84%)	1083 (82.4%)	
1–3	110 (5.6%)	97 (5.1%)		74 (5.6%)	62 (4.7%)	
4–10	102 (5.2%)	102 (5.3%)		43 (3.2%)	46 (3.5%)	
> 10	213 (10.9%)	251 (13.1%)		96 (7.2%)	124 (9.4%)	
Piles of dog/cat feces in courtyard, one or more	3 (0.2%)	2 (0.1%)	1.47 (0.24, 8.78)	4 (0.3%)	6 (0.5%)	0.65 (0.18, 2.32)
Dried cow patties (goita) in courtyard, one or more	104 (5.3%)	109 (5.7%)	0.97 (0.72, 1.22)	129 (9.7%)	166 (12.6%)	0.77 (0.62, 0.95)

^a^Prevalence ratio for binary mediators; count ratio for count mediators; odds ratio for ordinal categorical mediators

^b^Estimate from ordinal logistic regression; reflects the odds ratio of being in a higher category (same estimate for each reference category) for participants in the sanitation vs. control arm

#### Effect Modification

In a prior analysis, we found that the intervention only reduced diarrheal disease during monsoon seasons and had no effect during dry seasons [5]. In a post-hoc analysis restricting to children living in index households, the intervention reduced diarrhea during monsoon seasons and marginally reduced it during dry seasons. Therefore, we conducted the mediation analysis separately by monsoon and dry season to capture potential seasonal differences in mediation pathways. We used daily rainfall data recorded by the Bangladesh Meteorological Department at three weather stations closest to the study region for the years 2014–2016 to define the monsoon season for each study year as the period between the first and last days of 10 mm or greater 5-day rolling average rainfall [16]. The monsoon seasons during this study period were April 2–September 27, 2014, March 31–September 25, 2015, and March 30–October 30, 2016.

#### Mediation Analysis

Analyses were initially masked and conducted as intent-to-treat. We pooled data across the eight rounds of data collection and matched outcome data to mediators reported in the same data collection round; this assumes that mediator status assessed at the same time as diarrhea is representative of mediator status prior to disease incubation, on average 1–5 days, [17] to establish temporal ordering. This assumption is likely valid for latrine quality indicators, which are unlikely to change over short periods, and latrine use practices, which we recorded as general behaviors without a specific recall period. Some feces management practices, however, were recorded over a short recent recall period. We recorded potty use in the last week and child feces disposal location and handling of the index child’s last feces. It is possible that these practices would change in response to a child’s case of diarrhea, resulting in reverse causation that would not be distinguishable in these results. To assess potential reverse causation for these mediators, we conducted a sensitivity analysis using mediator values measured one round (approximately 3 months) prior to the measurement of diarrhea. Because these mediator measurements precede diarrhea events, the temporal sequence required for causation is assured. Assuming potty use, child feces disposal location, and child feces handling practices are consistent between rounds (independent of active diarrheal cases), differences between models using concurrent vs. temporally ordered mediator and diarrhea measurements could indicate reverse causation when mediators were measured simultaneously with diarrhea. We limited this sensitivity analysis to survey records in which the season (monsoon or dry) of a given data collection round matched that of the previous round, so diarrhea and mediator status were measured during the same season.

**Fig. 1:**
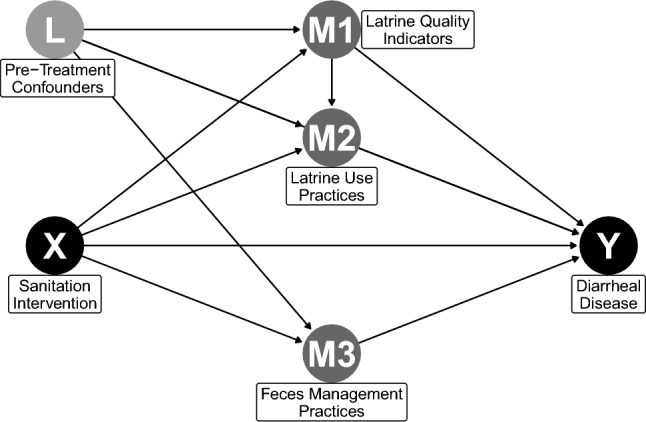
Directed acyclic graph (DAG) of causal pathways between random assignment into the sanitation intervention or control arms of the WASH Benefits trial (X) and child diarrheal disease (Y). Latrine quality (M1) and feces management practices (M3) are assumed to be independent of other mediators. Latrine use practices (M2) are hypothesized to be affected by latrine quality indicators, resulting in post-treatment confounding of mediating pathways. Pre-treatment confounders (L) are confounders of mediator-outcome associations

We followed the AGReMA Statement on reporting mediation analyses [12]. We employed the counterfactual-based framework for causal mediation analysis developed by Imai et al. and made available in the *mediation* package (version 4.5.0) in R [18–20]. Traditional approaches to mediation analysis, such as path analysis or structural equation modeling, require estimating mediation effects through all pathways in one model [21]. However, Imai et al. show that the traditional approach does not produce any benefits for causal inference compared to successive modeling of one mediator at a time and that both methods assume that mediators are causally independent from one another [19]. We assumed that latrine quality indicators and feces management practices were not caused by other mediators and assessed mediation successively for those variables. In contrast, we hypothesized that latrine use practices may be causally dependent on latrine quality indicators (Fig. 1). For example, randomization into the intervention arm might directly increase latrine use due to behavioral promotion, while also influencing latrine use indirectly through increased access to higher quality latrines. This dependence might produce post-treatment confounding of the latrine use-diarrhea relationship by latrine quality indicators, which violates necessary assumptions of independence for the estimation of mediation effects using the standard approach of Imai et al. [20, 22]. However, Imai et al. further developed an approach for estimating mediation effects of causally dependent mediators based on varying-coefficient linear structural equation models [19]. We used this approach for multiple mediation to assess mediation through latrine use practices, accounting for latrine quality.

The mediation analysis was conducted in three steps. In the first step, each potential mediator was regressed on intervention assignment (sanitation vs. control) to determine if the intervention had a significant effect on the potential mediator. Possible intervention effects included increases in desired latrine features or behaviors (e.g., more potty use among intervention recipients compared to controls) and reductions in undesired behaviors (e.g., less open defecation among intervention recipients compared to controls), both of which were hypothesized to reduce diarrhea. We fit binomial or modified Poisson (if binomial models did not converge) models for binary mediators, Poisson models for countable mediators, and ordinal logistic regression models for ordinal categorical mediators. We estimated robust standard errors to account for cluster-randomization and repeated measures and adjusted for study block to account for geographical matching. The mediation analysis required a consistent number of observations in all three steps, thus step one models were restricted to survey records without missing values for diarrheal disease or covariates included in step two. Only potential mediators that were statistically associated with intervention arm (alpha = 0.05) were retained for the second and third steps.

In the second step, diarrheal disease was regressed on intervention assignment (sanitation vs. control), controlling for the potential mediator and presumed mediator-outcome confounders. This step assessed whether the effect of the intervention on diarrheal disease changed after controlling for the mediator of interest and whether the mediator was associated with diarrheal disease controlling for intervention arm. We fit binomial or modified Poisson (if binomial models did not converge) models with robust standard errors and adjusting for study block. A set of potential confounding covariates were considered based on plausible causal mechanisms. Exposure-outcome confounders would be variables that cause diarrhea and be associated with the exposure, which in this study was a randomized intervention. We previously found that randomization led to good balance between study arms in this sample [23], suggesting unintended variables did not influence study arm assignment. Mediator-outcome confounders would be independent causes of diarrhea that are also associated with a given mediator. Adjusting for mediator-outcome confounders is necessary for this approach to mediation analysis [19]. Additionally, adjusting for causal predictors of diarrheal disease, even if they are not associated with a given mediator, can increase statistical precision due to the prospective study design [24]. The same set of potential mediator-outcome confounders was considered for all potential mediators, including: child’s age and sex, mother’s age, number of children under 18 years old in the household, number of individuals living in the compound, time to the household’s primary drinking water source, housing materials (improved vs. unimproved roof, floor, and walls), mother’s education level, food insecurity (using the Household Food Insecurity Access Scale), and wealth (from principal component analysis using 21 household assets). Potential covariates were measured once at the baseline of the parent trial, except child’s age, which was updated for each survey round. We used a statistical approach to filter relevant covariates to avoid unnecessarily increasing the dimensionality of our models. Potential covariates were included if they were statistically associated with diarrheal disease (alpha = 0.20) in bivariate regression, run separately by season.

In the third step for latrine quality indicators and feces management practices, causal mediation was assessed for each potential mediator that was retained in step one using the *mediate* function in the *mediation* package in R [20]. For each potential mediator, the results of models from steps one and two were used to estimate mediation effects. Specifically, we estimated the average causal mediation effect (ACME), defined as the effect of intervention assignment on diarrheal disease that was caused by the intervention’s effect on the mediator of interest and the mediator’s subsequent effect on diarrheal disease, and the average direct effect (ADE), defined as the effect of intervention assignment on diarrheal disease independent of the pathway through the mediating variable. ACMEs and ADEs were estimated as prevalence differences (PDs) between children in the sanitation and control arms of the trial. A negative ACME indicates that the intervention effect enacted through a given mediator was protective against diarrhea, either by increasing a desired mediating variable (e.g., potty use) or reducing an undesired mediating variable (e.g., open defecation). Confidence intervals and p-values were estimated using 1000 quasi-Bayesian Monte Carlo simulations [20]. Clustered standard errors were estimated with study block as the unit of clustering. In the third step for latrine use practices, we used the *multimed* function in the *mediation* package to estimate ACMEs and ADEs accounting for latrine quality indicators that were statistically significant mediators [20]. Confidence intervals were estimated using nonparametric bootstrapping on 1000 simulations [20]. Unlike other methods of mediation analysis, the approach developed by Imai et al. allows for interaction between treatment (the intervention arm) and the mediator [18, 19]. We report average estimates of the ACME and ADE, calculated as the weighted mean of conditional estimates for intervention and control households, with weights equal to the proportion of each group [20].

Because the intervention was designed to impact all potential mediators simultaneously, it is expected that the mediators are correlated. By analyzing correlated mediators in separate models, it is possible to estimate ACMEs whose sum is greater than the total observed effect [21]. Imai et al. have shown that these estimates are unbiased under assumptions of independence that this randomized trial meets [19]. To assess the overlap of potential mediators, e.g., the degree to which their independent mediation effects may reflect a shared process, we estimated correlation coefficients between mediators that were found to have statistically significant ACMEs, separately by season. We estimated Pearson’s correlation coefficients between binary variables; for ordinal variables, we estimated polychoric correlations, which assumes each ordinal variable represents a normally distributed latent variable and maintains Pearson’s correlation scale (− 1, 1) for comparability.

## Results

We enrolled 720 compounds (360 from the sanitation arm and 360 from the control arm) from the parent trial into the longitudinal substudy. Eighty percent of participants were available for all eight visits, and 96% provided data during six or more visits. Loss to follow-up was similar in the sanitation and control arms, and compounds that were lost to follow-up were similar to those with complete follow-up [23]. We collected a total of 3872 survey records (1956 sanitation; 1916 control) during monsoon seasons and 2643 records (1328 sanitation; 1315 control) during dry seasons from a total of 1079 individual children (535 sanitation; 544 control) under five living in index households. Ten records (3 sanitation; 7 control) were missing diarrheal disease information and were excluded from regression models. An additional 39 records (16 sanitation; 23 control) from 11 individual children (4 sanitation; 7 control) were excluded due to missing covariates (age and/or sex), resulting in a final sample size of 3850 records in monsoon seasons and 2626 records in dry seasons. Final sample sizes for each mediation model are reported in Table S1.

The prevalence of diarrheal disease among children in index households was 12.4% (10.0% sanitation; 14.8% control) during monsoon seasons and 13.0% (11.8% sanitation; 14.2% control) during dry seasons. The intervention reduced diarrheal disease by 32% (5 percentage points) in index households during monsoons seasons (prevalence ratio (PR) = 0.68, 95% CI 0.54, 0.86; prevalence difference (PD) = − 0.045, 95% CI − 0.074, − 0.016) and marginally reduced diarrhea by 17% (2 percentage points) during dry seasons (PR = 0.83, 95% CI 0.64, 1.09; PD = − 0.023, 95% CI − 0.055, 0.010).

Results from the first step of the mediation analysis indicate that all latrine quality indicators were significantly improved by the intervention (alpha = 0.05), although many latrine quality indicators were also high in the control arm (Table 1). For example, 76% of control index households primarily used a hygienic latrine (defined as an improved latrine that was observed to safely contain feces), 96% a latrine with a slab, and 96% a latrine with improved flooring, compared to 98%, 99%, and 99% among intervention recipients, respectively. A majority of latrine use and feces management practices were also improved among intervention recipients (Table 1). Among control households, 10% reported that men ever practiced open defecation and about 4% reported open defecation by women compared to just over 1% and < 1% for men and women in intervention households, respectively. Child open defecation was less common in the intervention arm compared to controls, but children under three were still reported to openly defecate sometimes in 76% of index households in the intervention arm compared to 91% among controls. However, potty use and safe management of child feces after open defecation was substantially improved. Index children reportedly used a potty for at least half of defecation events in 94% of intervention households vs. 17% of controls. Among intervention recipients, 76% reported disposing of child feces in a latrine compared to 23% of controls, and among intervention recipients where children openly defecated, 70% reported disposing of child feces in a latrine compared to 17% of controls. In contrast, sani-scoop or similar tool use to pick up child feces was less common among intervention recipients (55%) than controls (72%).

Step two of the mediation analyses showed significant associations between potential mediators retained from step one and diarrheal disease, adjusting for intervention arm and potential confounders (Table S2). Approximately 30% of survey records (representing at least one record from 95% of study households) had two successive data collection rounds that occurred in the same season and were used for the sensitivity analysis with time-ordered mediator and diarrhea measurements. For feces management practices that may have been susceptible to reverse causation (potty use, child feces disposal location, and child feces handling methods), associations with diarrheal disease were similar when re-estimated using mediator values measured one round prior to diarrhea, suggesting minimal impacts of reverse causation (Table S3). Only one mediator (child feces disposal location) that was significantly associated with diarrhea during dry seasons in the original model was no longer associated with diarrhea during dry seasons in sensitivity models.

**Fig. 2:**
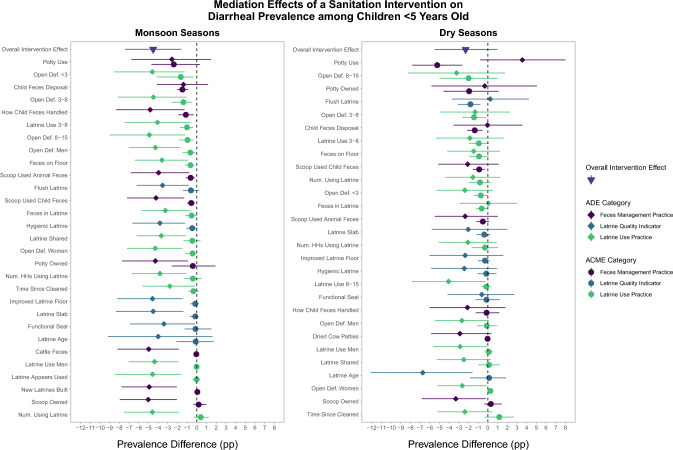
Mediation effects of the sanitation intervention on diarrheal prevalence among children < 5 years by latrine quality indicators, latrine use practices, and feces management practices. Effects are presented separately for monsoon and dry seasons. Average Causal Mediation Effects (ACME; circles) and Average Direct Effects (ADE; diamonds) are plotted together for each potential mediator (y-axis) along with the overall effect of the intervention (triangle). ACMEs are the effects of the sanitation intervention on childhood diarrheal disease that were achieved by the intervention’s effect on each mediator. ADEs are the residual effect of the intervention through other pathways after accounting for the mediator. Mediation effects are shown as prevalence difference estimates in percentage points (x-axis). Categories of potential mediators are indicated by color. Mediators are ordered from largest (top) to smallest (bottom) ACME by season; mediators are not aligned between seasons

Step three of the mediation analysis found that intervention effects on diarrhea were primarily mediated through latrine use practices and feces management practices (Fig. 2), although the magnitude of ACMEs for individual mediators were small compared to ADEs, suggesting that each mediator on its own only explained a small part of the intervention effect (Table S1). Mediators that were statistically significant during both monsoon and dry seasons included visible feces in the pit of the primary latrine, open defecation by children aged 3–8 years, open defecation by children aged < 3 years, where the index child’s most recent feces were disposed (e.g., latrine or environment), and sani-scoop or other tool use to pick up child feces. Mediators with statistically significant effects during monsoon seasons alone included presence of feces on the floor of the primary latrine, children aged 3–8 years always/usually using latrine for defecation, open defecation by men, women, and children aged 8–15 years, how caregivers handled the index child’s most recent feces (e.g., tool or paper), and sani-scoop or other tool use to pick up animal feces. Mediators significant during dry seasons alone included whether the primary latrine was a flush/pour-flush facility and potty use by the index child. The strongest mediation effects (listed in order of largest ACMEs) for the reduction in diarrhea during monsoon seasons were through reduced open defecation among children < 3 years (PD = − 0.017, 95% CI − 0.044, − 0.003), increased safe disposal of the index child’s most recent feces (PD = − 0.015, 95% CI − 0.021, − 0.009), reduced open defecation among children 3–8 years (PD = − 0.014, 95% CI − 0.025, − 0.005), and increased safe handling of the index child’s most recent feces (PD = − 0.012, 95% CI − 0.020, − 0.003). The strongest mediation effects during dry seasons were through increased potty use by the index child (PD = − 0.052, 95% CI − 0.078, − 0.025), flush/pour-flush primary latrine (PD = − 0.018, 95% CI − 0.029, − 0.008), reduced open defecation among children 3–8 years (PD = − 0.014, 95% CI − 0.027, − 0.002), and increased safe disposal of the index child’s most recent feces (PD = − 0.014, 95% CI − 0.021, − 0.006).

Across mediation models, estimated ACMEs summed to values greater than the observed total effect in each season, likely due to collinearity between mediators that were each targeted by the intervention. However, correlations between statistically significant mediators were generally weak (Tables S1 and S2). Among 66 correlations between 12 statistically significant mediators in monsoon seasons, we estimated seven correlation coefficients with absolute values greater than 0.5 (range − 0.59, 0.65) between indicators of open defecation, how child feces were handled, and where child feces were disposed (Table S4). Among 21 correlations between seven statistically significant mediators in dry seasons, we estimated eight correlation coefficients with absolute values greater than 0.5 (range − 0.76, 0.65) between indicators of open defecation among children, where child feces were disposed, sani-scoop or other tool use for child feces, potty use, and access to a flush/pour-flush latrine (Table S5). Moderate to strong correlations between some mediators likely reflect shared mechanisms of mediation that cannot be separately estimated, such as the intervention’s simultaneous effects on improving child potty use and reducing open defecation by children. However, no correlations were strong enough to suggest complete collinearity between related variables.

## Discussion

We found that the WASH Benefits Bangladesh sanitation intervention successfully improved latrine quality, latrine use, and safe feces management among targeted households, while the intervention’s effect on diarrheal disease among children under five was primarily mediated through improved latrine use and child feces management rather than improved latrine quality. Strongest mediators (highest ACMEs) were those related to child feces management, including potty use, safe child feces disposal, and reduced open defecation among children. ACMEs for individual mediators generally were small (i.e., PDs ranging between 0.5 and 2 percentage points), and direct effects (ADEs) conditional on a single mediation pathway accounted for most of the intervention’s total effects. However, each of our models is causally independent of other models and, in theory, ACMEs could be summed over a set of mediators to calculate their total mediation effect. In practice, model estimates include overlapping effects due to the correlated nature of simultaneous intervention targets, and their sum would double count this shared effect. Individual estimates of mediation effects are difficult to interpret in isolation. The results of this analysis are best interpreted along with attention to qualitative patterns and suggest that improvements in child feces management were the strongest mediators and, collectively, may account for a substantial portion of the intervention’s effects.

These results highlight the importance of addressing child feces management for preventing pathogen transmission, despite receiving insufficient attention in sanitation programs until recent years [9]. Although many studies do not differentiate open defecation between adults and children [25], open defecation is much more common among young children than other age groups, even when latrine access is high [9]. Young children are also more likely to defecate within the domestic environment, such as in the courtyard in this study, while adults who openly defecate often do so away from home [9, 26]. Risks from child open defecation are compounded by common misconceptions that child feces are harmless or less dangerous than adult feces, which contributes to poor or no disposal of child feces that are left in the home environment [4, 9, 11, 26–29]. Consequently, feces of young children are a larger contributor to fecal contamination in domestic settings than feces of adults and older children [30].

The intervention resulted in reduced child open defecation, but the practice remained common even among intervention households (76% vs. 91% of controls). Intervention households were substantially more likely to dispose of child feces in a latrine compared to controls both among all households (76% vs. 23%) and specifically among households with open defecating children (70% vs. 17%). Index children also much more likely to use a potty for at least half of defecation events (94% vs. 14% of controls). We note that sani-scoop or other tool use to pick up child feces was lower among intervention recipients than controls (55% vs. 72%), which reflects increased potty use and reduced open defecation, thus fewer feces to pick up, for intervention recipients. The results of this study suggest that aiming for disposal of child feces in a latrine may be a more responsive target for intervention programs compared to preventing child open defecation, although even the relatively modest reductions in child open defecation were the strongest mediator of the intervention’s effects on diarrheal disease in the analysis. Additionally, even when child feces are safely disposed of following open defecation, contamination remains on floors even after cleaning [31]. These results indicate that there is significant room for improvement in the effectiveness of behavioral interventions in reducing child open defecation, and that achieving greater reductions in child open defecation could significantly improve child health. Sanitation programs should develop and assess strategies aimed at achieving larger reductions in child open defecation.

Additional latrine use practices that mediated reductions in diarrhea included increased latrine use among children under three, reduced open defecation among all age groups, improved latrine cleanliness (lack of feces on floor), and reduced presence of visible feces in the pit. Although we expected presence of visible feces in the pit to be an indicator of increased latrine use, fewer households in the sanitation arm had visible feces in the pit compared to controls. Observed feces in the pit may reflect fuller pits and poorer containment in the control arm, which is likely associated with latrine age, pit depth, and pit emptying practices. We note that latrine access and use among adults was high and open defecation by adults was infrequent even among controls, suggesting that cultural norms favoring latrine use were already in place and the intervention may have been primed to close gaps in latrine use behaviors rather than form new habits from scratch. Mediation results indicate that additional reductions in open defecation achieved by the intervention were instrumental in reducing child diarrhea in this setting. These findings are consistent with a recent modeling study in Mozambique indicating that deposition of even a small amount of fecal material into the environment can sustain ongoing enteric infections despite latrine access [32].

We previously hypothesized that the intervention’s effects were likely achieved through improved latrine quality based on prevalent latrine use norms in the setting [5]. However, despite improved latrine quality being associated with reduced diarrheal disease in this analysis, latrine quality indicators did not mediate reductions in diarrhea, except for mediation by access to a flush/pour-flush latrine in the dry season (Fig. 2). The intervention successfully improved all latrine quality indicators we assessed, reaching nearly universal coverage with hygienic latrines (improved latrines observed to safely contain feces) in the sanitation arm, compared to 76% of controls. At the first round of this substudy, latrines in the sanitation arm were more likely to be flush/pour flush (99% vs. 76%) and have a functional water seal (95% vs. 46%) than controls while over 95% of latrines in both arms had slabs [5]. Given the high existing access to on-site latrines, it is possible that the intervention’s potential for improving latrine quality was too restricted to significantly mediate diarrheal effects. It is also possible that the measured indicators did not fully capture true latrine quality or the salient latrine characteristics that drive interruptions in pathogens transmission, and high latrine quality may have reduced diarrheal disease through unidentified pathways.

To our knowledge, this study is the first causal mediation analysis of a sanitation intervention. Previously, a mediation analysis of a combined handwashing and water quality intervention in urban Bangladesh assessed psychosocial factors as potential mediators between the intervention and behavior change, as measured by stored drinking water quality and observed handwashing with soap [15]. Thus, while this study focused on sanitation quality and behaviors as potential mediators of health outcomes, George et al. assessed the upstream mediating pathways that led to behavioral changes. This difference highlights the various uses of mediation analysis in evaluating water, sanitation, and hygiene interventions, including identifying the important features of a multi-component intervention or identifying specific psychosocial mechanisms through which an intervention leads to behavior change.

Strengths of the study included repeated measurement of outcomes and potential mediators in eight data collection rounds over approximately 2.5 years, which allowed us to capture any temporal variation and assess mediation separately across monsoon and dry seasons while maintaining a high sample size. We were also able to take advantage of recent advances in methods for causal mediation analysis, which allowed us to isolate mediation effects that are causally dependent on other mediators. A potential limitation of the study was the simultaneous measurement of mediators and outcomes during the same data collection round. For feces management practices that were measured with short recall (e.g., how the child’s most recent stool was managed), concurrent measurement can lead to reverse causation since these practices may change in response to a child experiencing diarrhea. However, a sensitivity analysis using mediators measured one round prior (about 3 months) to diarrhea outcomes found minimal differences in the associations between these mediators and diarrhea (Table S3). Only one mediator (child feces disposal location during dry seasons) was no longer associated with diarrheal disease in sensitivity models. Increased disposal of child feces into latrines during diarrheal episodes could explain that variable’s significant mediation effect during dry seasons in models with concurrent mediator and diarrhea measurements, although the mediator-diarrhea association during monsoon seasons remained significant regardless of measurement order. Safer feces disposal during diarrheal episodes would also be a potential benefit of the intervention that may have reduced pathogen transmission in subsequent weeks, which were not measured in this study. Another limitation was that most indicators of latrine use and feces management practices were self-reported while latrine quality indicators were observed by field staff. Likewise, diarrheal disease in children was reported by caregivers, although the intervention’s effects on diarrhea are supported by similarly protective effects on objectively measured enteric infections and by biologically plausible seasonal effects on diarrheal disease [4–7]. The use of self-reported mediators and a caregiver-reported outcome could potentially result in spuriously high mediation effects by these pathways. Finally, the relatively high number of mediators assessed in this study increased the risk of estimating spurious effects due to multiple testing. However, if spurious mediation effects were estimated for some individual variables, it remains unlikely that the overall findings based on groups of variables, particularly those related to child feces management, were driven by chance findings alone.

The WASH Benefits Bangladesh trial successfully reduced the prevalence of diarrheal disease among children through a multi-component sanitation intervention, setting it apart from almost all latrine-based sanitation interventions evaluated to date. In this analysis, we found that reduced open defecation by young children and improved management of child feces were the primary mediators of the intervention’s effects, despite the relatively low uptake of the delivered child feces management tools [29]. Child feces are rarely a focus of sanitation interventions. Interventions that effectively improve safe management of young children’s feces, especially where latrine access and other sanitation norms are already in place, may result in increased benefits to child health.

## Supplementary Information

Below is the link to the electronic supplementary material.

Supplementary file 1 (DOCX 40 kb)

## Data Availability

The datasets generated for this research are available on OSF, along with a pre-specified analysis plan and data analysis scripts: https://osf.io/6u7cn/.

## Abbreviations

ACME: Average causal mediation effect
ADE: Average direct effect
PD: Prevalence difference
PR: Prevalence ratio
CI: Confidence interval
DAG: Directed acyclic graph
WSH or WASH: Water, sanitation, and hygiene
icddr,b: International Centre for Diarrhoeal Disease Research, Bangladesh
CLTS: Community-led total sanitation
AGReMA: A guideline for reporting mediation analyses
