# Supplementary material for: Improved Child Feces Management Mediates Reductions in Childhood Diarrhea from an On-Site Sanitation Intervention: Causal Mediation Analysis of a Cluster-Randomized Trial in Rural Bangladesh
Source: J Epidemiol Glob Health. 2024 Mar 20;14(3):765–78. doi: 10.1007/s44197-024-00210-y (PMC11444030; doi:10.1007/s44197-024-00210-y)
Supplement: Supplementary file 1 — (DOCX 40 kb) [file 44197_2024_210_MOESM1_ESM.docx]

**Supplementary Information**

**Improved child feces management mediates reductions in childhood diarrhea from an on-site sanitation intervention: causal mediation analysis of a cluster-randomized trial in rural Bangladesh**

Jesse Contreras, Mahfuza Islam, Andrew Mertens, Amy J. Pickering, Benjamin F. Arnold, Jade Benjamin-Chung, Alan E. Hubbard, Mahbubur Rahman, Leanne Unicomb, Stephen P. Luby, John M. Colford, Jr., Ayse Ercumen*

*Corresponding Author: Ayse Ercumen, Jordan Hall Addition 2225, Raleigh, NC, 27606,

+1 (510) 225 8828, aercume@ncsu.edu

| **Table S1** Estimation of mediation effects by season (mediation analysis step three). Average causal mediation effects (ACME) and average direct effects (ADE) are reported as absolute prevalence differences for diarrheal disease among children under five between the sanitation and control arm. Statistically significant ACMEs and ADEs (alpha = 0.05) are bolded. Total intervention effect (sum of ACME and ADE) varies by mediator due to different covariates and sample sizes | | | | | | |
| --- | --- | --- | --- | --- | --- | --- |
|  | Monsoon Season ^a^ | | | Dry Season ^b^ | | |
| Mediator | Sample Size | ACME  Sanitation vs. Control  (95% CI) | ADE  Sanitation vs. Control  (95% CI) | Sample Size | ACME  Sanitation vs. Control (95% CI) | ADE  Sanitation vs. Control (95% CI) |
| *Latrine Quality Indicators* |  |  |  |  |  |  |
| Primary latrine used by index household is hygienic | 3,768 | -0.46% (-1.11, 0. 20) | **-3.70% (-6.40, -0.95)** | 2,588 | -0.13% (-1.04, 0.91) | -2.42% (-5.39, 1.14) |
| Primary latrine is flush or pour-flush | 3,763 | -0.61% (-1.42, 0.23) | **-3.49% (-6.04, -0.99)** | 2,583 | **-1.75% (-2.87, -0.75)** | 0.27% (-3.48, 4.37) |
| Primary latrine has functional water seal | 3,178 | -0.16% (-1.36, 1.32) | **-3.38% (-6.68, -0.13)** | 2,207 | -0.14% (-1.33, 1.29) | -0.80% (-4.49, 2.72) |
| Primary latrine has slab | 3,769 | -0.17% (-0.72, 0.17) | **-4.54% (-8.36, -1.54)** | 2,588 | -0.34% (-1.12, 0.32) | -2.10% (-6.04, 2.23) |
| Primary latrine has improved floor materials | 3,768 | -0.17% (-0.69, 0.16) | **-4.75% (-8.97, -1.61)** | 2,588 | -0.32% (-1.08, 0.20) | -2.19% (-6.10, 1.92) |
| Age of primary latrine (recorded in rounds 1-2 only) | 813 | -0.12% (-2.11, 2.00) | -3.98% (-9.18, 2.00) | 915 | 0.19% (-1.60, 2.00) | **-6.80% (-11.45, -2.00)** |
| *Latrine Use Practices* |  |  |  |  |  |  |
| Primary latrine is shared with other households | 3,769 | -0.47% (-1.32, 0.41) | **-3.81% (-6.60, -1.10)** | 2,583 | 0.12% (-1.01, 1.24) ^c^ | -2.48% (-5.32, 0.35) ^c^ |
| Number of households primary latrine shared with | 3,769 | -0.37% (-1.31, 0.46) | **-3.77% (-6.84, -0.95)** | 2,583 | -0.31% (-1.60, 0.98) ^c^ | -2.06% (-4.94, 0.82) ^c^ |
| Number of people who use primary latrine | 3,769 | 0.39% (-0.37, 1.14) | **-4.51% (-7.19, -1.94)** | 2,583 | -0.82% (-1.98, 0.35) ^c^ | -1.55% (-4.39, 1.28) ^c^ |
| Visible feces in pit of primary latrine | 3,182 | **-0.56% (-1.24, 0.00)** | **-3.19% (-5.50, -0.71)** | 2,202 | **-0.64% (-1.17, -0.11) ^c^** | 0.05% (-2.88, 2.97) ^c^ |
| Primary latrine appears to be used | 3,769 | 0.00% (-0.40, 0.34) | **-4.67% (-8.41, -1.60)** | NA | NA | NA |
| Feces on floor in primary latrine | 3,769 | **-0.65% (-1.26, -0. 27)** | **-3.52% (-6.00 -0.79)** | 2,583 | -0.91% (-1.89, 0.06) ^c^ | -1.47% (-4.24, 1.30) ^c^ |
| Time since primary latrine last cleaned | 2,731 | -0.38% (-0.93, 0.20) | **-2.73% (-5.37, -0.20)** | 2,366 | 1.18% (-0.32, 2.68) ^c^ | -2.37% (-5.34, 0.59) ^c^ |
| Men in household always/usually use latrine for defecation | 3,621 | -0.03% (-0.32, 0.25) | **-4.49% (-7.40, -1.68)** | 2,459 | 0.10% (-0.37, 0.57) ^c^ | **-2.86% (-5.51, -0.22) ^c^** |
| Children 8-15 in household always/usually use latrine for defecation | NA | NA | NA | 1,209 | -0.16% (-0.64, 0.32) ^c^ | **-4.06% (-7.91, -0.21) ^c^** |
| Children 3-8 in household always/usually use latrine for defecation | 2,397 | **-0.99% (-1.77, -0.35)** | **-4.00% (-7.45, -0.71)** | 1,560 | -0.93% (-1.92, 0.05) ^c^ | -1.85% (-5.36, 1.66) ^c^ |
| Men in household ever practice open defecation | 3,698 | **-0.64% (-1.36, -0.16)** | **-4.21% (-6.92, -1.56)** | 2,467 | -0.12% (-1.11, 0.87) ^c^ | -2.68% (-5.48, 0.11) ^c^ |
| Women in household ever practice open defecation | 3,843 | **-0.45% (-1.27, -0.01)** | **-4.40% (-7.03, -1.66)** | 2,582 | 0.26% (-0.04, 0.56) ^c^ | -2.64% (-5.29, 0.01) ^c^ |
| Children 8-15 in household ever practice open defecation | 1,843 | **-0.97% (-1.92, -0.35)** | **-4.81% (-9.03, -0.90)** | 1,160 | -1.99% (-5.06, 1.07) ^c^ | -3.23% (-8.07, 1.62) ^c^ |
| Children 3-8 in household ever practice open defecation | 2,427 | **-1.39% (-2.47, -0.45)** | **-4.58% (-8.34, -0. 83)** | 1,537 | **-1.43% (-2.69, -0.17) ^c^** | -1.32% (-4.93, 2.28) ^c^ |
| Children under 3 in household ever practice open defecation | 3,259 | **-1.71% (-4.43, -0.32)** | **-4.62% (-8.58, -1.18)** | 2,282 | **-0.73% (-1.42, -0.03) ^c^** | -2.38% (-5.35, 0.58) ^c^ |
| *Feces Management Practices* |  |  |  |  |  |  |
| New latrine built within compound since previous visit | 3,008 | 0.05% (-0.15, 0.22) | **-4.87% (-7.84, -1.95)** | NA | NA | NA |
| Safer disposal of index child’s most recent feces | 3,833 | **-1.49% (-2.08, -0.92)** | -1.33% (-4.38, 1.06) | 2,620 | **-1.36% (-2.09, -0.56)** | -0.12% (-3.76, 3.14) |
| Potty, scoop, or tool used to handle index child’s most recent feces | 3,082 | **-1.15% (-1.96, -0.28)** | **-4.78% (-8.44, -1.00)** | 2,113 | -0.11% (-1.22, 1.23) | -2.17% (-5.80, 1.68) |
| Owns child potty | 3,843 | -0.40% (-2.40, 1.99) | **-4.36% (-7.65, -1.09)** | 2,623 | -2.04% (-4.70, 1.05) | -0.29% (-5.45, 5.12) |
| Potty use by index child in past week | 3,159 | -2.29% (-4.84, 0.55) | -2.69% (-7.30, 1.13) | 2,176 | **-5.18% (-7.83, -0.0248)** | 3.40% (-1.31, 8.26) |
| Owns sani-scoop | 3,843 | 0.20% (-0.36, 0.98) | **-5.01% (-8.04, -2.03)** | 2,623 | 0.30% (-0.39, 0.016) | **-3.25% (-6.62, -0.08)** |
| Sani-scoop or other tool generally used to pick up child feces | 3,843 | **-0.59% (-1.05, -0.18)** | **-4.20% (-7.20, -1.33)** | 2,623 | **-0.89% (-1.53, -0.0033)** | -2.02% (-4.93, 1.04) |
| Sani-scoop or other tool generally used to pick up animal feces | 3,843 | **-0.65% (-1.18, -0.20)** | **-4.01% (-7.06, -1.30)** | 2,623 | -0.52% (-1.19, 0.08) | -2.40% (-5.54, 0.83) |
| Number of piles of cattle feces in courtyard | 3,843 | -0.05% (-0.20, 0.03) | **-4.89% (-8.16, -1.79)** | NA | NA | NA |
| Cow patties (goita) in courtyard, one or more | NA | NA | NA | 2,623 | -0.03% (-0.21, 0.09) | -2.95% (-5.94, 0.35) |
| ^a^ Adjusted for improved household roof, age of child in days, and sex of child  ^b^ Adjusted for improved household walls, food insecurity, wealth, age of child in days, and sex of child  ^c^ Multiple mediator analysis used to account for significant latrine quality mediator (flush/pour-flush latrine) | | | | | | |

| **Table S2** Relationships between potential mediators and diarrheal disease among children under five, adjusted for intervention treatment, and residual effect of the intervention on diarrheal disease, accounting for the mediator (mediation analysis step two). Independent variables for each model were intervention arm, the potential mediator, and potential confounders of the mediator and diarrheal disease | | | | |
| --- | --- | --- | --- | --- |
|  | Monsoon Season ^a^ | | Dry Season ^b^ | |
| Mediator | Adjusted Association between Mediator and Diarrheal Disease  PR (95% CI) | Residual Intervention Effect on Diarrheal Disease  PR (95% CI) | Adjusted Association between Mediator and Diarrheal Disease  PR (95% CI) | Residual Intervention Effect on Diarrheal Disease  PR (95% CI) |
|  | Mediator | Sanitation vs. Control Arm | Mediator | Sanitation vs. Control Arm |
| Unadjusted (Reference) | -- | 0.68 (0.54, 0.86) | -- | 0.83 (0.64, 1.09) |
| *Latrine Quality Indicators* |  |  |  |  |
| Primary latrine used by index household is hygienic | 0.82 (0.65, 1.04) | 0.74 (0.62, 0.89) | 0.94 (0.70, 1.26) | 0.83 (0.67, 1.03) |
| Primary latrine is flush or pour-flush | 0.84 (0.67, 1.06) | 0.75 (0.62, 0.91) | 0.63 (0.48, 0.84) | 1.02 (0.79, 1.30) |
| Primary latrine has functional water seal | 0.96 (0.74, 1.23) | 0.75 (0.60, 0.93) | 0.96 (0.70, 1.33) | 0.94 (0.72, 1.23) |
| Primary latrine has slab | 0.62 (0.39, 0.98) | 0.72 (0.61, 0.86) | 0.46 (0.30, 0.71) | 0.87 (0.71, 1.07) |
| Primary latrine has improved floor materials | 0.61 (0.39, 0.96) | 0.72 (0.61, 0.86) | 0.50 (0.31, 0.80) | 0.86 (0.70, 1.06) |
| Age of primary latrine, one year increase (recorded in rounds 1-2 only) | 1.00 (0.97, 1.04) | 0.80 (0.57, 1.11) | 1.00 (0.96, 1.04) | 0.63 (0.45, 0.88) |
| *Latrine Use Practices* |  |  |  |  |
| Primary latrine is shared with other households | 1.12 (0.93, 1.35) | 0.73 (0.61, 0.88) | 0.99 (0.79, 1.24) | 1.01 (0.78, 1.32) |
| Number of households primary latrine shared with, one additional household | 1.03 (0.97, 1.10) | 0.73 (0.61, 0.88) | 1.02 (0.93, 1.11) | 1.03 (0.79, 1.35) |
| Number of people who use primary latrine, one additional person | 0.98 (0.95, 1.01) | 0.68 (0.57, 0.82) | 1.02 (0.99, 1.06) | 1.07 (0.82, 1.38) |
| Visible feces in pit of primary latrine | 1.22 (1.00, 1.48) | 0.76 (0.63, 0.93) | 1.23 (0.97, 1.57) | 1.06 (0.82, 1.37) |
| Primary latrine appears to be used | 0.80 (0.32, 2.02) | 0.71 (0.60, 0.84) | NA | NA |
| Feces on floor in primary latrine | 1.77 (1.35, 2.33) | 0.74 (0.62, 0.89) | 1.39 (1.02, 1.88) | 1.05 (0.82, 1.35) |
| Time since primary latrine last cleaned, one additional day | 1.01 (1.00, 1.02) | 0.76 (0.60, 0.97) | 1.00 (0.98, 1.01) | 1.00 (0.77, 1.31) |
| Men in household always/usually use latrine for defecation | 0.93 (0.64, 1.36) | 0.70 (0.58, 0.83) | 1.19 (0.67, 2.10) | 0.99 (0.77, 1.27) |
| Children 8-15 in household always/usually use latrine for defecation | NA | NA | 0.73 (0.42, 1.26) | 0.89 (0.63, 1.26) |
| Children 3-8 in household always/usually use latrine for defecation | 0.66 (0.52, 0.84) | 0.70 (0.55, 0.88) | 0.67 (0.51, 0.88) | 1.10 (0.79, 1.52) |
| Men in household ever practice open defecation | 1.60 (2.22, 2.10) | 0.71 (0.59, 0.85) | 1.11 (0.73, 1.68) | 0.99 (0.77, 1.27) |
| Women in household ever practice open defecation | 1.74 (1.19, 2.53) | 0.70 (0.59, 0.84) | 0.28 (0.04, 2.12) | 1.01 (0.78, 1.29) |
| Children 8-15 in household ever practice open defecation | 1.88 (1.30, 2.72) | 0.67 (0.52, 0.87) | 2.49 (1.60, 3.86) | 0.86 (0.61, 1.22) |
| Children 3-8 in household ever practice open defecation | 1.46 (1.17, 1.83) | 0.68 (0.54, 0.86) | 1.50 (1.13, 1.99) | 1.09 (0.78, 1.52) |
| Children under 3 in household ever practice open defecation | 1.75 (1.21, 2.55) | 0.74 (0.62, 0.89) | 1.95 (1.28, 2.95) | 1.06 (0.81, 1.37) |
| *Feces Management Practices* |  |  |  |  |
| New latrine built within compound since previous visit | 0.85 (0.53, 1.37) | 0.64 (0.52, 0.79) | NA | NA |
| What was done with index child’s most recent feces ^c^  *Left there in courtyard*  *Thrown into environment or garbage*  *Used or put into latrine* | 0.79 (0.72, 0.87) | 0.88 (0.72, 1.08) | 0.83 (0.74, 0.92) | 1.00 (0.79, 1.27) |
| How was index child’s feces handled  *Used potty, sani-scoop, or other instrument*  *Used hands, cloth, paper, leaves, straw (REF)* | 0.75 (0.61, 0.92) | 0.69 (0.56, 0.85) | 0.97 (0.75, 1.24) | 0.85 (0.68, 1.08) |
| Owns child potty | 0.95 (0.75, 1.19) | 0.71 (0.60, 0.85) | 0.77 (0.55, 1.08) | 0.97 (0.69, 1.35) |
| Potty use by index child in past week ^c^  *Don’t own or never use*  *Used to use potty, but no longer*  *Less than half the time*  *More than half the time*  *Every time* | 0.91 (0.83, 1.00) | 0.80 (0.59, 1.08) | 0.82 (0.72, 0.92) | 1.35 (0.93, 1.96) |
| Owns sani-scoop | 1.11 (0.78, 1.58) | 0.67 (0.57, 0.80) | 1.15 (0.74, 1.79) | 0.79 (0.64, 0.96) |
| Sani-scoop or other tool used to pick up child feces | 1.33 (1.10, 1.61) | 0.71 (0.60, 0.85) | 1.37 (1.10, 1.71) | 0.86 (0.70, 1.05) |
| Sani-scoop or other tool used to pick up animal feces | 0.73 (0.59, 0.89) | 0.73 (0.61, 0.87) | 0.80 (0.63, 1.03) | 0.84 (0.68, 1.03) |
| Number of piles of cattle feces in courtyard ^c^  *0*  *1-2*  *3-10*  *>10* | 1.05 (0.96, 1.15) | 0.68 (0.58, 0.81) | NA | NA |
| Cow patties (goita) in courtyard, one or more | NA | NA | 1.08 (0.81, 1.44) | 0.80 (0.66, 0.98) |
| ^a^ Adjusted for improved household roof, age of child in days, and sex of child  ^b^ Adjusted for improved household walls, food insecurity, wealth, age of child in days, and sex of child  ^c^ Mediation analysis required ordinal categorical mediators to be modeled as continuous; estimates reflect a change from one category to the next highest category | | | | |

| **Table S3** Sensitivity analysis for mediators potentially susceptible to reverse causation. Adjusted relationship between mediators and diarrheal disease, with mediators measured in two ways: 1) mediator and diarrheal disease were measured in the same round (identical to Table S1) and 2) mediators measured in the previous round relative to diarrhea. Sensitivity models include only survey records in which diarrheal disease and mediators were measured during the same season (monsoon or dry) | | | | |
| --- | --- | --- | --- | --- |
|  | Monsoon Season ^a^ | | Dry Season ^b^ | |
| Mediator | Adjusted Association between Mediator and Diarrheal Disease  PR (95% CI) | | Adjusted Association between Mediator and Diarrheal Disease  PR (95% CI) | |
| *Feces Management Practices* | Mediator and Diarrheal Disease Measured in Same Round | Mediator Measured One Round (~3 months) Prior to Diarrheal Disease | Mediator and Diarrheal Disease Measured in Same Round | Mediator Measured One Round (~3 months) Prior to Diarrheal Disease |
| Total number of child records available | 3,865 | 1,384 | 2,640 | 617 |
| What was done with index child’s most recent feces ^c^  *Left there in courtyard*  *Thrown into environment or garbage*  *Used or put into latrine* | 0.79 (0.72, 0.87) | 0.86 (0.74, 0.99) | 0.83 (0.74, 0.92) | 0.95 (0.74, 1.23) |
| How was index child’s feces handled  *Used potty, sani-scoop, or other instrument*  *Used hands, cloth, paper, leaves, straw (REF)* | 0.75 (0.61, 0.92) | 0.65 (0.44, 0.98) | 0.97 (0.75, 1.24) | 1.02 (0.67, 1.86) |
| Potty use by index child in past week ^c^  *Don’t own or never use*  *Used to use potty, but no longer*  *Less than half the time*  *More than half the time*  *Every time* | 0.91 (0.83, 1.00) | 0.88 (0.74, 1.05) | 0.82 (0.72, 0.92) | 0.67 (0.56, 0.80) |
| ^a^ Adjusted for intervention arm, improved household roof, age of child in days, and sex of child  ^b^ Adjusted for intervention arm, improved household walls, food insecurity, wealth, age of child in days, and sex of child  ^c^ Mediation analysis required ordinal categorical mediators to be modeled as continuous; estimates reflect a change from one category to the next highest category | | | | |

| **Table S4** Correlation matrix between statistically significant mediators during monsoon seasons. Correlations between binary variables were estimated with Pearson correlation coefficients. Correlations including ordinal variables (*“What was done with index child’s most recent feces?”*) were estimated with polychoric correlation coefficients. Correlations with an absolute value greater than 0.5 are bolded | | | | | | | | | | | | | | | | | | |
| --- | --- | --- | --- | --- | --- | --- | --- | --- | --- | --- | --- | --- | --- | --- | --- | --- | --- | --- |
| Mediator | Visible feces in pit of primary latrine | Feces on floor in primary latrine | Children 3-8 in household always/usually use latrine for defecation | Men in household ever practice open defecation | Women in household ever practice open defecation | Children 8-15 in household ever practice open defecation | Children 3-8 in household ever practice open defecation | | Children under 3 in household ever practice open defecation | | What was done with index child’s most recent feces^a^ | | | How was index child’s feces handled | | Sani-scoop or other tool used to pick up child feces | | Sani-scoop or other tool used to pick up animal feces |
| Visible feces in pit of primary latrine | 1.000 |  |  |  |  |  |  | |  | |  | | |  | |  | |  |
| Feces on floor in primary latrine | 0.101 | 1.000 |  |  |  |  |  | |  | |  | | |  | |  | |  |
| Children 3-8 in household always/usually use latrine for defecation | -0.084 | -0.119 | 1.000 |  |  |  |  | |  | |  | | |  | |  | |  |
| Men in household ever practice open defecation | 0.053 | 0.140 | -0.151 | 1.000 |  |  |  | |  | |  | | |  | |  | |  |
| Women in household ever practice open defecation | 0.048 | 0.102 | -0.054 | **0.523** | 1.000 |  |  | |  | |  | | |  | |  | |  |
| Children 8-15 in household ever practice open defecation | 0.047 | 0.240 | -0.213 | **0.554** | **0.523** | 1.000 |  | |  | |  | | |  | |  | |  |
| Children 3-8 in household ever practice open defecation | 0.150 | 0.114 | **-0.592** | 0.246 | 0.192 | 0.252 | 1.000 | |  | |  | | |  | |  | |  |
| Children under 3 in household ever practice open defecation | 0.080 | 0.044 | -0.169 | 0.081 | 0.045 | 0.046 | 0.192 | | 1.000 | |  | | |  | |  | |  |
| Safer disposal of index child’s most recent feces^a^ | -0.322 | -0.313 | 0.372 | -0.415 | -0.446 | -0.408 | **-0.578** | | **-0.591** | | 1.000 | | |  | |  | |  |
| How was index child’s feces handled | -0.097 | -0.154 | 0.117 | -0.154 | -0.087 | -0.207 | -0.196 | | -0.212 | | **0.648** | | | 1.000 | |  | |  |
| Sani-scoop or other tool generally used to pick up child feces | 0.083 | 0.037 | -0.151 | 0.021 | 0.021 | 0.072 | 0.304 | | 0.403 | | -0.414 | | | 0.000 | | 1.000 | |  |
| Sani-scoop or other tool generally used to pick up animal feces | -0.073 | -0.084 | 0.089 | -0.094 | -0.078 | -0.092 | -0.079 | | -0.041 | | 0.208 | | | 0.097 | | 0.111 | | 1.000 |
| ^a^ Correlations estimated with polychoric correlation coefficients for ordinal variable | | | | | | | |  | |  | |  |  | |  | |  | |

| **Table S5:** Correlation matrix between statistically significant mediators during dry seasons. Correlations between binary variables were estimated with Pearson correlation coefficients. Correlations including ordinal variables (*“What was done with index child’s most recent feces”* and *“Potty use by index child in past week”*) were estimated with polychoric correlation coefficients. Correlations with an absolute value greater than 0.5 are bolded. | | | | | | | |
| --- | --- | --- | --- | --- | --- | --- | --- |
| Mediator | Primary latrine is flush or pour-flush | Visible feces in pit of primary latrine | Children 3-8 in household ever practice open defecation | Children under 3 in household ever practice open defecation | What was done with index child’s most recent feces^a^ | Potty use by index child in past week^a^ | Sani-scoop or other tool used to pick up child feces |
| Primary latrine is flush or pour-flush | 1.000 |  |  |  |  |  |  |
| Visible feces in pit of primary latrine | 0.184 | 1.000 |  |  |  |  |  |
| Children 3-8 in household ever practice open defecation | -0.233 | 0.094 | 1.000 |  |  |  |  |
| Children under 3 in household ever practice open defecation | -0.153 | 0.093 | 0.147 | 1.000 |  |  |  |
| Safer disposal of index child’s most recent feces | **0.505** | -0.239 | **-0.538** | **-0.623** | 1.000 |  |  |
| Potty use by index child in past week^a^ | **0.654** | -0.182 | **-0.574** | **-0.759** | **0.629** | 1.000 |  |
| Sani-scoop or other tool generally used to pick up child feces | -0.150 | 0.078 | 0.321 | 0.431 | -0.449 | **-0.594** | 1.000 |
| ^a^ Correlations estimated with polychoric correlation coefficients for ordinal variable | | | | | | | |
